# Circadian Clock Genes Act as Diagnostic and Prognostic Biomarkers of Glioma: Clinic Implications for Chronotherapy

**DOI:** 10.1155/2022/9774879

**Published:** 2022-07-04

**Authors:** Ruihuan Chai, Min Liao, Ling Ou, Qian Tang, Youfang Liang, Nan Li, Wei Huang, Xiao Wang, Kai Zheng, Shaoxiang Wang

**Affiliations:** ^1^School of Pharmaceutical Sciences, Shenzhen University Health Science Center, Shenzhen 518000, China; ^2^Bacteriology & Antibacterial Resistance Surveillance Laboratory, Shenzhen Institute of Respiratory Diseases, Shenzhen People's Hospital (The Second Clinical Medical College, Jinan University, Shenzhen 518020, China; ^3^The First Affiliated Hospital, Southern University of Science and Technology, Shenzhen 518020, China; ^4^School of Pharmacy, Jinan University, Guangzhou 510630, China; ^5^Department of Pharmacy, Shenzhen People's Hospital (The Second Clinical Medical College, Jinan University, Shenzhen 518020, China

## Abstract

Gliomas are the most common primary intracranial tumors and closely related to circadian clock. Due to the high mortality and morbidity of gliomas, exploring novel diagnostic and early prognostic markers is necessary. Circadian clock genes (CCGs) play important roles in regulating the daily oscillation of biological processes and the development of tumor. Therefore, we explored the influences that the oscillations of circadian clock genes (CCGs) on diagnosis and prognosis of gliomas using bioinformatics. In this work, we systematically analyzed the rhythmic expression of CCGs in brain and found that some CCGs had strong rhythmic expression; the expression levels were significantly different between day and night. Four CCGs (*ARNTL*, *NPAS2*, *CRY2*, and *DBP*) with rhythmic expression were not only identified as differentially expressed genes but also had significant independent prognostic ability in the overall survival of glioma patients and were highly correlated with glioma prognosis in COX analysis. Besides, we found that CCG-based predictive model demonstrated higher predictive accuracy than that of the traditional grade-based model; this new prediction model can greatly improve the accuracy of glioma prognosis. Importantly, based on the four CCGs' circadian oscillations, we revealed that patients sampled at night had higher predictive ability. This may help detect glioma as early as possible, leading to early cancer intervention. In addition, we explored the mechanism of CCGs affecting the prognosis of glioma. CCGs regulated the cell cycle, DNA damage, Wnt, mTOR, and MAPK signaling pathways. In addition, it also affects prognosis through gene coexpression and immune infiltration. Importantly, *ARNTL* can rhythmically modulated the cellular sensitivity to clinic drugs, temozolomide. The optimal point of temozolomide administration should be when *ARNTL* expression is highest, that is, the effect is better at night. In summary, our study provided a basis for optimizing clinical dosing regimens and chronotherapy for glioma. The four key CCGs can serve as potential diagnostic and prognostic biomarkers for glioma patients, and *ARNTL* also has obvious advantages in the direction of glioma chronotherapy.

## 1. Introduction

Glioma is the most common primary intracranial tumor, accounting for more than 70% of malignant brain tumors, and is associated with high mortality and morbidity [1]. Gliomas commonly occur in the cerebellum, brainstem, and cortex [2, 3] and are currently classified into low-grade glioma (LGG, grade II and III) and glioblastoma (GBM, grade IV) [4]. Notably, a considerable proportion of LGG develops rapidly in a short time and deteriorates into GBM. Analysis of the isocitrate dehydrogenase status and 1p/19q codeletion can contribute to personalized diagnosis and prognosis [5]. Despite these findings, the poor prognosis of glioma still urgently requires the discovery of novel biomarkers for the early detection of glioma and to improve patient survival.

The circadian clock is a molecular timekeeping mechanism that exists in all creatures and regulates the daily oscillations of biological processes and behavior [6]. Circadian clock genes (CCGs) form feedback loops to maintain normal function of the oscillating system and to keep it in sync with the environmental cycle. To date, 13 CCGs have been well identified, including *ARNTL/BMAL1* [7], *CLOCK* [8], *CRY1*, *CRY2* [9], *DBP*, *NPAS2* [10], *NR1D1*, *NR1D2*, *PER1*, *PER2*, *PER3* [11], *RORA* [12], and *TIMELESS* [13]. Increasing evidence has revealed that CCGs play critical roles in DNA damage and repair, cell proliferation and metastasis, immunity, and tumorigenesis [14, 15]. Other studies have shown that intervening with the circadian clock could effectively influence stem cell growth, tumor aggressiveness, and drug delivery to improve therapeutic outcomes [16–19]. For instance, activation of *PER1* effectively inhibited the progression of pancreatic cancer [20]. *NR1D2* promoted the proliferation, migration, and invasion of GBM cells through specific targets [21]. Although a recent study showed that CCGs have significant prognosis value in glioma [22], the influence of CCGs rhythmic fluctuations on prognosis has not been conclusively determined. Therefore, our study focused on the effect of genes rhythm on glioma prognosis.

In this study, we systematically analyzed the circadian expression patterns of CCGs in brain tissues. The differential expression analysis was used to identify possible diagnostic markers. Kaplan–Meier survival curves, Cox proportional hazards model, and nomogram were used to assess the prognostic value of CCGs. Besides, CCG-involved cancer-related pathways and coexpression network analysis were also launched. Furthermore, CCG-related cellular sensitivity to temozolomide was demonstrated, which may facilitate glioma chronotherapy.

## 2. Materials and Methods

### 2.1. Datasets and Data Availability

Profiles of patients with glioma were downloaded from The Cancer Genome Atlas (TCGA) and the Chinese Glioma Genome Atlas (CGGA). TCGA is a project launched by US in 2006 that contains data on various human cancers, while CGGA is a biobank of glioma samples for the Chinese population. A total of 698 cases from TCGA and 1,018 cases from the CGGA were collected. Glioma patients with missing OS values or OS < 30 days were excluded to reduce statistical bias in our analysis. The circadian rhythm data of CCGs in brain tissues of mice were collected from GSE54652 of the GEO database.

### 2.2. Pan-Cancer Analysis and Gene Correlation Analysis

GSCA (genomic cancer analysis platform) was used for pan-cancer analysis. The Sangerbox was used to show coexpression patterns among CCGs in brain tissues. The clock correlation distance (CCD), an algorithm that have been constructed in the literature [23], was used here to infer circadian clock progression in a group of samples based on the coexpression of 13 clock genes. Gene expression data were obtained from GSE54652 and TCGA. Red notes indicate that the effect of high expression on survival risk is high and vice versa is indicated by the green notes. Pearson's correlation was used to assess the internal relationship of CCGs.

### 2.3. CCG Rhythmic Expression in Mouse Brain Tissues

CCG expression data in the brain tissue downloaded from GEO's GES54652. This dataset was collected from different organs of mice at two hourly intervals over a 48-hour period. Microarray data were used to show daily changes in gene expression. To determine if the data is rhythmic, R language package called “JTK_CYCLE” was used to detect the rhythm of each gene. The JTK_CYCLE package, with parameters set to fit time-series data to exactly 24 h periodic waveforms and statistical analysis, was used. When *P* < 0.05, the gene was considered to be rhythmically expressed [24]. GraphPad Prism (v7.0) was used to draw graphs representing cyclical fluctuations. The rhythm graphs of the CCGs were also constructed using GraphPad Prism.

### 2.4. Differential Expression Analysis

Gene expression profiles of brain tissues in glioma patients with clinical grade were extracted from two datasets (698 tumor samples from TCGA and 1,018 tumor samples from the CGGA). Gene expression levels were normalized using the edgeR and limma packages for TCGA and CGGA data [25, 26], respectively, in R software. *P* value < 0.05 was considered differentially expressed genes (DEGs).

### 2.5. Analysis of Methylation, Copy Number Variation (CNV), and Single-Nucleotide Variation (SNV)

GSCA was used to reveal the role of CNV, methylation, and SNV in the regulation of CCG expression. Significance was set at FDR ≤ 0.05. The Pearson correlation coefficient was used to analyze the correlation between CNV, methylation, and gene expression.

### 2.6. Survival Analysis

The CCG expression in patient tumor samples from the two datasets with survival data (TCGA and CGGA) was included in the survival analysis. Survival curves were plotted by the Kaplan–Meier method using R software, and the median value of gene expression was set as the group cut off separating the high-expression and low-expression groups. Univariate and multivariate Cox regression analyses were calculated by “survival” package. The hazard ratio (HR) and *P* value of each DEG of CCGs were determined based on gene expression and overall survival of patients using the univariate Cox regression model with the survival package in R software. HR > 1 and HR < 1 indicate that the higher expression of the gene is associated with worse or better overall survival, respectively.

### 2.7. Prediction Models

R software was used to create Cox proportional hazards prediction models based on CCG expression levels and overall survival. Accordingly, the following formula determines the risk score of each patient:

(1) $$\begin{array}{c} \text{Risk score}=\overset{n}{\underset{i=1}{\sum }}{\text{Coef}}_{i}\times {\text{Exp}}_{i}, \end{array}$$

where *n* is the number of involved genes, Coef is the coefficient of every gene, and Exp is the expression level (log2). Patients were divided into the high-risk and low-risk groups according to their risk scores. Receiver operating characteristic (ROC) curves were presented to show the ability to predict survival based on the risk scores by R software, and area under the curve (AUC) values represented the accuracy of predicting survival. Nomograms have the ability to predict survival according to age, grade, and CCG prediction model using R software (the expression level is corrected by log2).

### 2.8. Pathway Activity and Functional Enrichment Analysis

The pathway activity of CCGs was analyzed using GSCA and GSEA. GSCA elucidated the relevant pathway of CCGs in pan-cancer. GSEA analysis was used to explore the relevant pathways associated with CCGs in glioma, including the hallmark gene set, KEGG gene set, and GO gene set. Patient samples from TCGA were divided into the high- and low-risk groups according to the risk scores based on CCG expression.

### 2.9. Coexpression Network Analysis

The WGCNA R package [27] was used to assess the correlation coefficients between the CCGs and other hub genes. The coexpressed genes with correlation > 0.5 were extracted. Cytoscape (v 3.8.0) visualized the correlation between key CGGs and coexpressed genes in the network.

### 2.10. Immune Infiltrate Analysis

Six immune cell types, B cells, CD4+ T cells, CD8+ T cells [28], neutrophils, dendritic cells (DCs) [29], and macrophages [30], in the tumor microenvironment have been previously reported. We used the Tumor Immune Estimation Resource (TIMER), an established algorithm, to estimate the correlation between immune cells and gene expression. Spearman's correlation and estimated statistical significance between CCG expression and immune cell signature infiltration are shown in scatter plots.

### 2.11. Drug Sensitivity Analysis

The correlation between key CCG expression and drug sensitivity was determined using GSCA. Drug sensitivity and gene expression profiling data of cancer cell lines were obtained from the Genomics of Drug Sensitivity in Cancer (GDSC). Spearman correlation was calculated based on the relationship between small molecule/drug sensitivity (IC_50_) and gene expression. The positive Spearman correlation means that the gene with high expression is resistant to the drug, vice versa.

### 2.12. Statistical Analysis

This study is based on a systematic purely bioinformatic analyses. Statistical analysis was performed using R (v4.0.3) and GraphPad Prism (v7.0.0). The HRs and 95% confidence intervals (CIs) were calculated to identify genes associated with overall survival. Pearson's correlation analysis was used to calculate the correlation coefficient. *P* value < 0.05 or FDR < 0.05 were considered statistically significant.

## 3. Results

### 3.1. Pan-Cancer Analysis and Coexpression Patterns of CCGs

**Figure 1:**
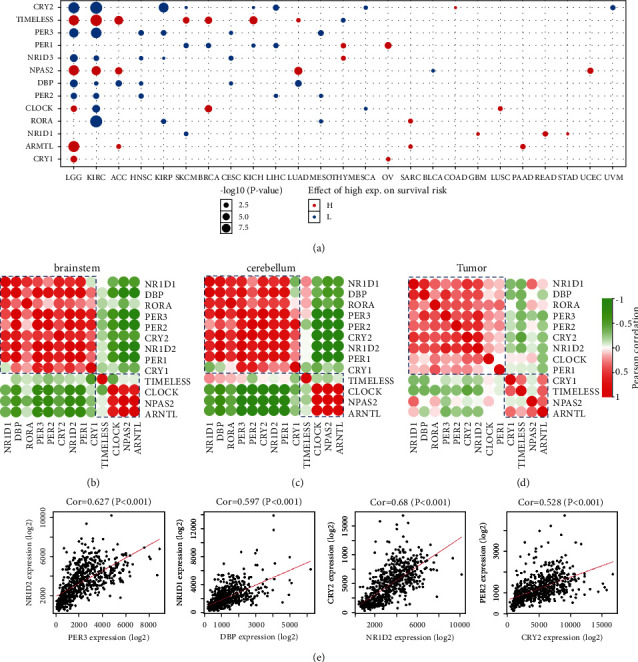
Pan-cancer analysis and coexpression patterns of CCGs. (a) Pan-cancer analysis of the effect of CCGs on survival risk. (b, c) Internal coexpression pattern of CCGs in normal brain tissues. (d) Internal coexpression pattern of CCGs in tumor tissues. (e) The scatter plots of correlation among genes in the same cluster in brain tissues.

The prognostic influence of CCGs on the survival risk of several cancers was performed (Figure 1(a)). LGG exhibited the highest *P* value in pan-cancer analysis, with 10 significant prognostic CCGs in total. The results indicated that among all cancers, glioma was affected mostly by CCGs. Next, we revealed the coexpression of CCGs in brain tissues (Figures 1(b)–1(d)), according to the coexpression correlations in brain, CCGs were apparently divided into two clusters: cluster I includes *ARNTL*, *NPAS2*, *CLOCK*, and *TIMELESS*, and another includes *CRY1*, *CRY2*, *DBP*, *NR1D1*, *NR1D2*, *PER1*, *PER2*, *PER3*, and *ROR*A. Similar coexpression relationship were found by using the CCD algorithm [23], indicating that the rhythmic oscillation patterns might be also alike in normal brain tissue and tumor samples. To further confirm the coexpression relationship in brain tissues, gene correlation maps were performed, which indicates that there is a high positive correlation between CCGs of the same cluster (Figure 1(e)).

### 3.2. Rhythmic Expression of CCGs in Brain Tissues

**Figure 2:**
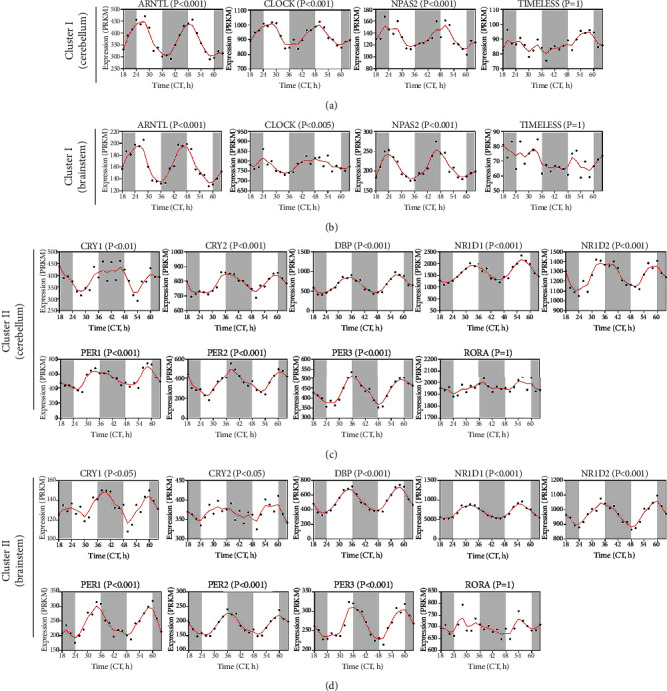
Circadian oscillations of CCGs in brain tissues. (a, b) Circadian oscillations of mouse cerebellum CCGs at different intervals. (c, d) Circadian oscillations of mouse brainstem CCGs at different intervals. The intersection of white and black background indicates the alternation of day and night.

Based on the day-night expression data of mouse genes in the GSE54652 dataset, we performed JTK analysis using bioinformatics methods in R software, in which *P* < 0.05 was considered a gene with rhythmic expression. The results showed the rhythmic expression of CCGs in consecutive 48 hours. Apparently, most of cluster CCGs exhibited a significant rhythmic expression pattern in the cerebellum (Figures 2(a) and 2(c)), and in brainstem (Figures 2(b) and 2(d)), respectively. The results covered two circadian cycles. There was little difference in CCG rhythmic expression between the cerebellum and brainstem, suggesting that CCGs rhythmic patterns in brain tissue are stable [31, 32]. The fluctuation patterns of the two clusters were opposite, with cluster I CCGs reaching the peak in the morning and cluster II CCGs reaching the peak in the evening.

### 3.3. CCGs Were Differentially Expressed in the Grade of Glioma

**Figure 3:**
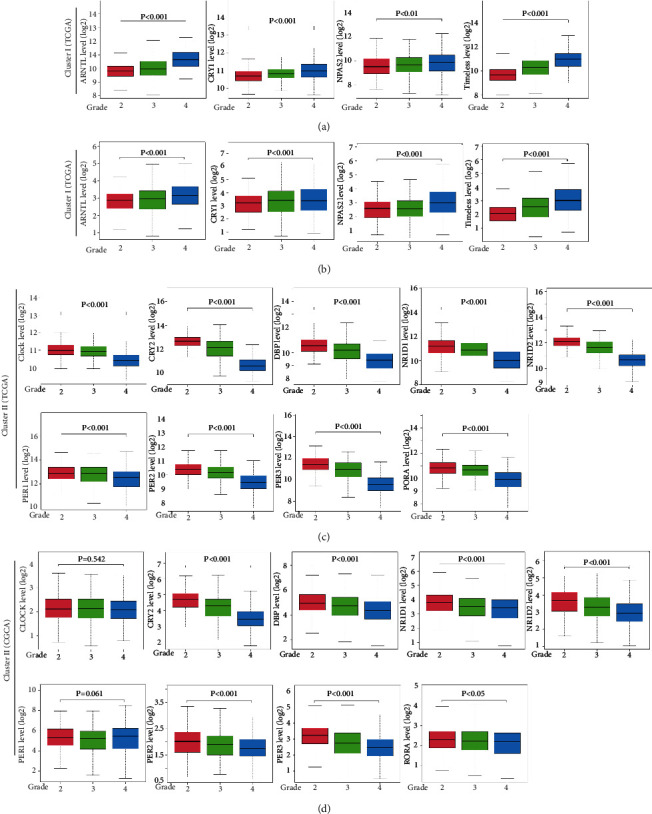
Differential expression of CCGs based on glioma grade. (a, b) High expression of cluster I CCGs in glioma grading in TCGA and CGGA. (c, d) Low expression of in cluster II CCGs in glioma grading in TCGA and CGGA.

**Figure 4:**
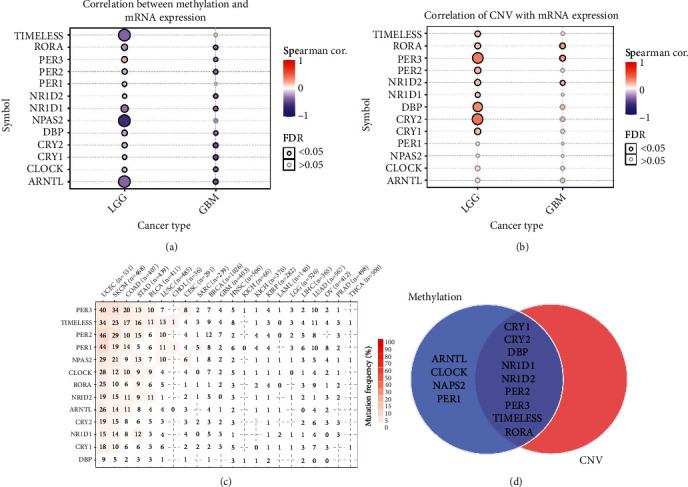
Correlations of DNA methylation, CNV, and SNP with CCGs. (a) Correlation between methylation and CCGs' mRNA expression. The size of the point represents the statistical significance, where the bigger the dot size, the higher the statistical significance. (b) Correlation of CNV with CCGs' mRNA expression. The size of the point represents the statistical significance, where the bigger the dot size, the higher the statistical significance. (c) SNP mutation frequency of CCGs in Pan cancer. (d) Graph of genes regulated by CNV and methylation. FDR: false discovery rate; cor: correlation.

To investigate the role of CCGs in all grades of glioma, including grade 2-4, we firstly analyzed the differential expression of CCGs in glioma grade and found that 11/13 CCGs, except for *CLOCK* and *PER1*, were recognized as DEGs in TCGA (Figures 3(a) and 3(b)) and CGGA (Figures 3(c) and 3(d)), respectively. Interestingly, two clusters' genes had opposite expression trends, which expression levels of cluster I and *CRY1* were increased with the grades, whereas cluster II and *CLOCK* decreased. We then examined the possible regulatory mechanism of CCG expression by analyzing methylation and copy number variation (CNV). All DEGs were negatively regulated by methylation (FDR < 0.05, Figure 4(a)), except for *PER3*. Besides, except for *PER1*, *NPAS2*, *CLOCK*, and *ARNTL*, the other 9 DEGs were positively regulated by copy number variation (FDR < 0.05, Figure 4(b)). The single-nucleotide variation of CCG was not observed in glioma (Figure 4(c)). The Venn diagram clearly demonstrated that 8 CCGs, including most cluster II CCGs, were regulated by both methylation and CNV (Figure 4(d)). The combination of methylation and CNV mediated most of the downregulation of cluster II CCGs. Collectively, these results implied that methylation and CNV mainly contributed to the differential expression of CCGs in gliomas.

### 3.4. CCGs as Prognostic Biomarkers for Glioma Patients

**Figure 5:**
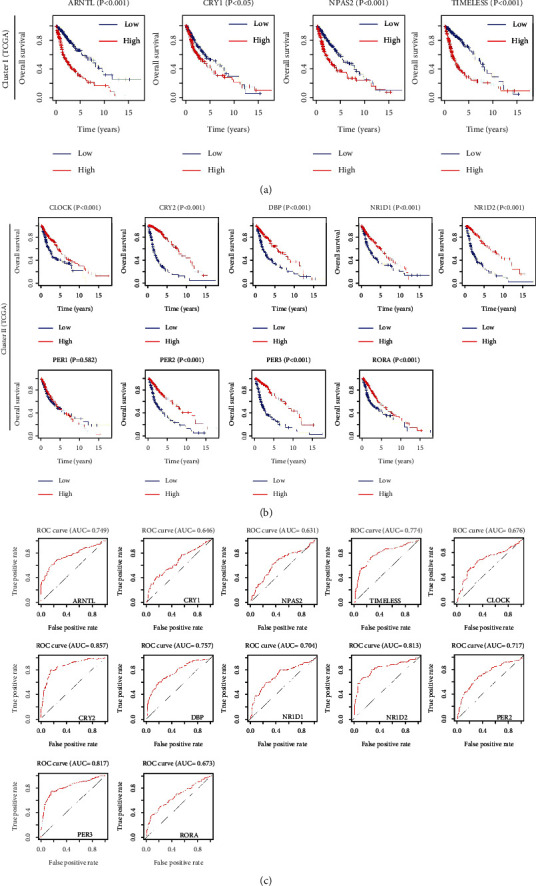
Survival analysis of CCGs in glioma patients from TCGA. (a) Kaplan–Meier survival curves of cluster I genes in TCGA. (b) Kaplan–Meier survival curves of cluster II genes in TCGA. (c) ROC curves of each CCG in glioma.

**Figure 6:**
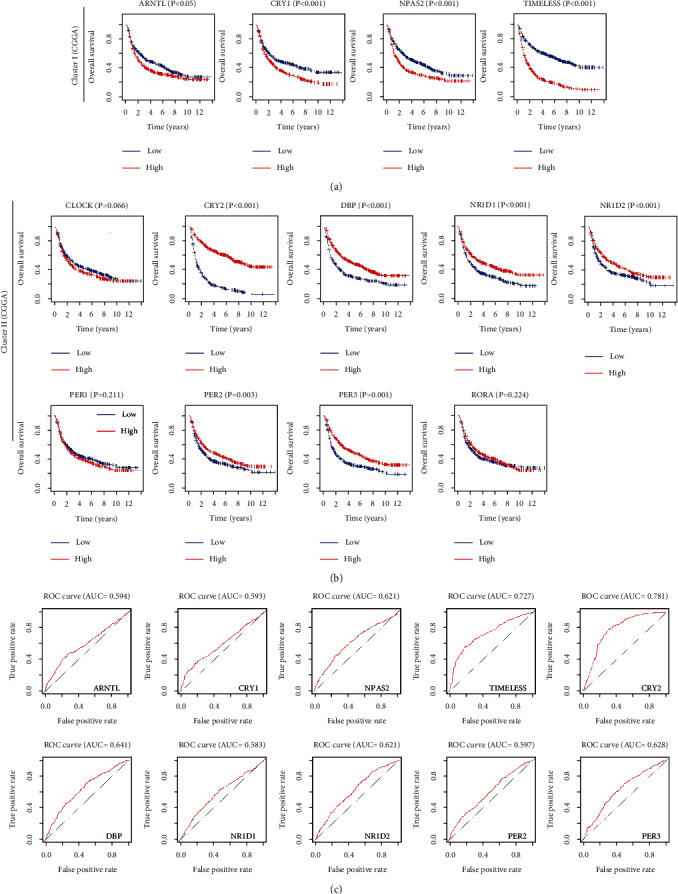
Survival analysis of CCGs in glioma patients from CGGA. (a) Kaplan–Meier survival curves of cluster I CCGs in CGGA. (b) Kaplan–Meier survival curves of cluster II CCGs in CGGA. (c) ROC curves of each CCGs in glioma.

The prognostic abilities of a single CCG were analyzed and survival curves showed that 10 differential expressed CCGs were significantly associated with the survival rates of glioma patients (*P* < 0.05). Importantly, high expression of cluster I and *CRY1* was associated with poor survival of glioma patients in both the TGCA and CGGA databases (Figures 5(a) and 6(a)). On the contrary, lower expression of cluster II and *CLOCK* was related to low survival time (Figures 5(b) and 6(b)). ROC curves were plotted to assess CCG predictive accuracy (AUC value ≥ 0.6 as predictive) [33], which showed that most of CCGs had accurate predictive ability in TCGA (Figure 5(c)) and CGGA (Figure 6(c)). Therefore, these CCGs were potential prognostic biomarkers for patients with glioma.

### 3.5. Prediction Model of CCGs by Cox Regression Analysis

**Figure 7:**
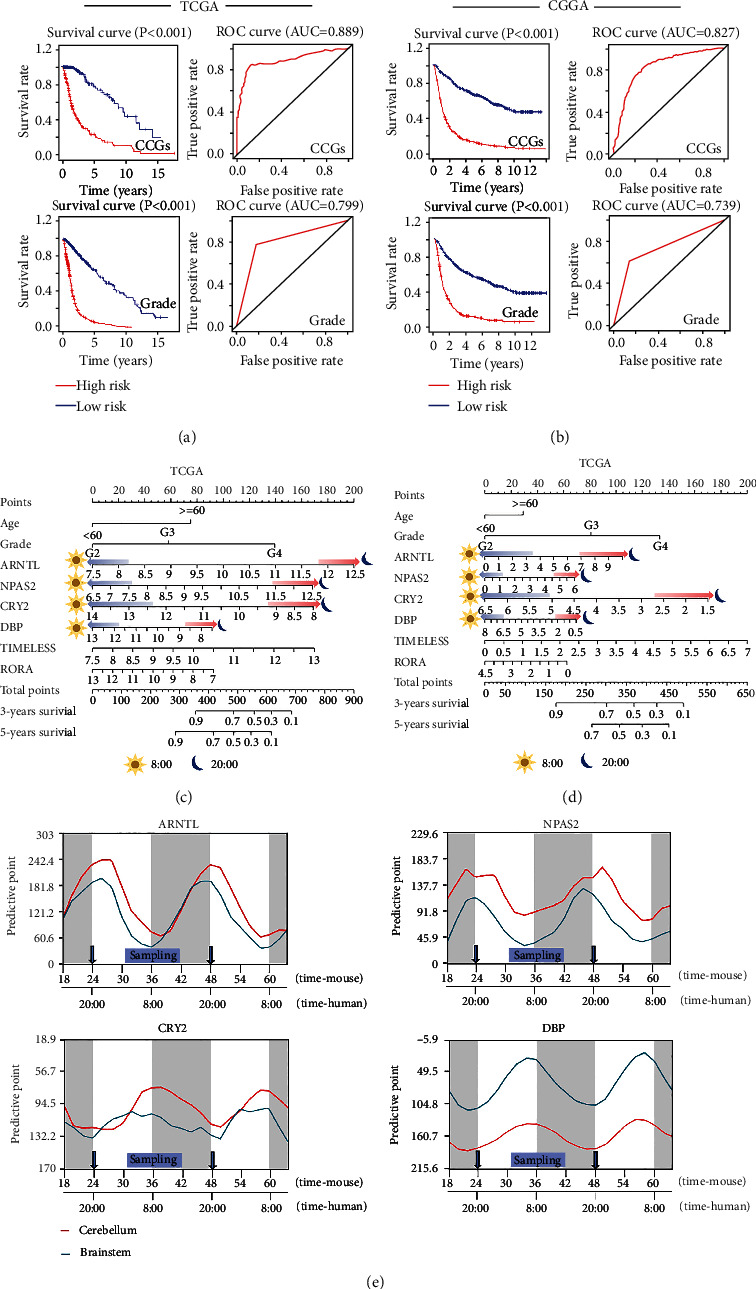
Prediction model of CCGs. (a, b) CCGs-based prediction model has higher accuracy than grade-based prediction model in glioma patients. (c, d) Nomogram showed risk scores in glioma patients increased in the evening and decreased in the morning. (e) The daily rhythmic fluctuations of key CCGs in human and mouse brain tissues were demonstrated in detail, and the black and white background indicates the passage of day and night.

Univariate and multivariate Cox regression analyses were performed to investigate the prognostic role of CCG combinations. As shown in Tables 1 and 2, univariate Cox regression analysis demonstrated that the poor survival of patients was related to grade, cluster I, and *CRY1*, with HR > 1 (HR > 1 represents a high-risk factor) [34], whereas 6 CCGs in cluster II, except for *PER1*, were considered low-risk factors. In addition, multivariate Cox regression analysis indicated that the combination of 6 CCGs, including *ARNTL*, *NPAS2*, *CRY2*, *DBP, RORA*, and *TIMELESS*, was significant for patient survival in both the TCGA and CGGA databases (Tables 1 and 2). Accordingly, a 6-CCG-based predictive model was constructed, and the survival curve and ROC curve clearly showed that the 6-CCG-based prediction model had higher accuracy than that of the prediction model based on grade in TCGA (Figure 7(a)) and CGGA (Figure 7(b)). The accuracy of ROC analysis was the similar for all grades of glioma (Fig S1). Furthermore, nomogram analysis was performed, which revealed that 4 of the 6 CCGs in combination, including oscillations of *ARNTL*, *NPAS2*, *CRY2*, and *DBP*, have significant impacts on the predictive accuracy of the 6-CCG-based predictive model (Figures 7(c) and 7(d)). Based on the finding that the expression peak phase of CCGs shifted by ~12 hours between the mouse and baboon [35], CCG expression fluctuations throughout the day in mice and humans, and appropriate sampling time were redisplayed (Figure 7(e)). For instance, *ARNTL* and *NPAS2* showed peak of expression in mouse in the morning, whereas in human, the peak phase occurred in beginning of the evening (about 8:00 pm). On the contrary, *CRY2* and *DBP* exhibited opposite expression patterns.

### 3.6. Pathway Enrichment Analysis of the Four Key CCGs

**Figure 8:**
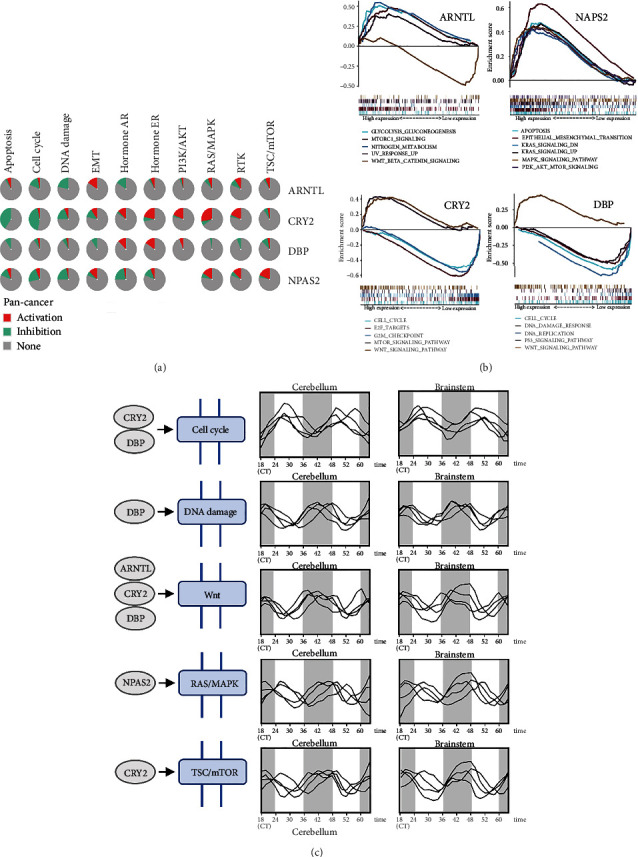
Pathway analysis of the four key CCGs. (a) The role of CCGs on cancer-related pathways in pan-cancer: red represents activation, green represents inhibition, and gray represents no effect. (b) Cancer pathways most associated with enrichment of key CCGs in glioma were showed. (c) In the above pathways, some genes showed rhythmic expression and showed close correlation with CCGs (FABP7, TTC28, FGFR2, PTPRE, DUSP11, FABP7, TEF, CIART, FMO2, SYSLTR2, GPR17, GJB6, ZBTB16, CALR, SFPQ, and SLC16A1).

GSCA and GSEA were further used to analyze the possible CCG-involved molecular mechanisms that the pie chart of GSCA demonstrated several pan-cancer pathways (Figure 8(a)), while GSEA showed key signaling pathways (Figure 8(b)). In most cases, cluster I genes (*ARNTL*, *NPAS2*) had opposite effects against cluster II genes (*CRY2*, *DBP*) in the same pathway. For instance, *ARNTL* activated Wnt signaling pathway which was inhibited by *CRY2* and *DBP.* Besides, cluster II mainly activated Wnt, mTOR pathway and inhibited cell cycle, DNA damage pathway to promote proliferation and tumor development. Moreover, key genes in these classic cancer-related pathways were found and their expression levels were analyzed, which also exhibited highly similar rhythmic fluctuations to CCGs (Figure 8C). Together, these results implied that CCGs regulated these cancer-related pathways in progression of glioma.

### 3.7. Coexpression Network Analysis of the Four Key CCGs

**Figure 9:**
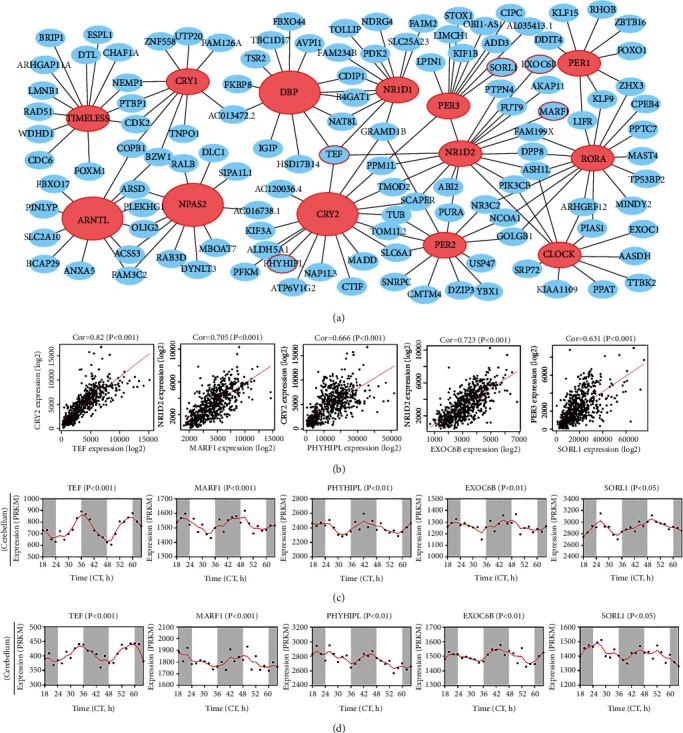
Coexpression network of the four key CCGs. (a) Coexpression network showed the relationship between key CCGs and related genes. (b) Scatter plots showed the high correlation between key CCGs and their closely coexpressed genes. (c, d) Rhythmic expression of closely coexpressed genes in the mouse cerebellum or in brainstem.

WGCNA was performed to identify the genes that were coexpressed with the four key CCGs (*ARNTL*, *NPAS2*, *CRY2*, and *DBP*) in glioma (Figure 9(a)). CCGs were displayed as red nodes and the coexpressed genes were marked as blue nodes. Besides, several genes having rhythmic expression were circled in red. It was found that cluster I and cluster II genes were tightly linked to each other, respectively. The correlations between the red circle genes (representative rhythmic genes) and CCGs were shown by scatter plots (Figure 9(b)). Correspondingly, these highly positively correlated genes also demonstrated rhythmic fluctuations in the cerebellum and brainstem (Figures 9(c) and 9(d)). Among them, *TEF* was tightly associated with *CRY2*, *DBP*, and *NR1D2* and thus had a similar rhythmic expression as cluster II genes. Other arrhythmic genes, such as *PDK2*, have prognostic abilities in lung adenocarcinoma [36]. Taken together, these findings suggested that CCGs can regulate the rhythm of other genes to interfere with the progression of glioma.

### 3.8. Immune Infiltrate Analysis of the Four Key CCGs

**Figure 10:**
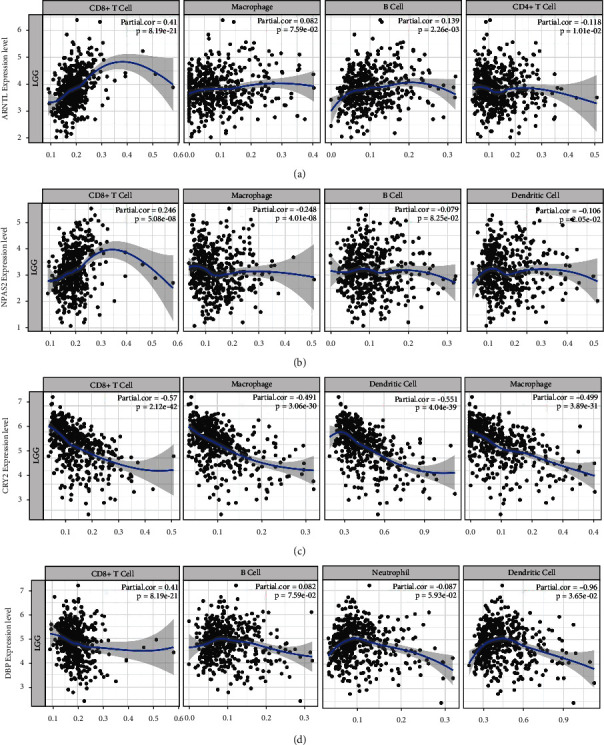
Correlation between immune infiltration and the four key CCGs. (a–d) *ARNTL*, *NPAS2*, *CRY2*, and *DBP* significantly correlated with different immune cells, including CD8+ T cell, CD4+ T cell, neutrophil, B cell, dendritic cell, and macrophage.

Immune infiltration is a pivotal biological characteristic of tumors. To investigate whether the prognosis of CCGs is related to immune infiltration, the correlations between these CCGs and immune cells in glioma were explored using the online tool TIMER. Four immune cell types with the close correlation to key CCGs (expression level adjusted by log2 TPM) were shown in Figure 10. The result showed that *ARNTL* and *NPAS2* was positively correlated with CD8+ T cells, whereas *CRY2* and *DBP* were negatively correlated with neutrophils and macrophages. This revealed that same cluster CCGs affect patient prognosis in a similar way in immune infiltration. *CRY2* is highly correlated with immune cells (|correlation| > 0.4), suggesting that *CRY2* may affect prognosis through immune infiltration more than other CCGs.  Table 3 showed the important internal biomarkers of immune cells, in which CCGs affect the state and function of immune cells through activation or inhibition of these biomarkers, thus affecting patient survival. Spearman's correlation in the table indicated that *CRY2* had prominent effect on these biomarkers.

### 3.9. Drug Sensitivity Analysis of the Four Key CCGs

**Figure 11:**
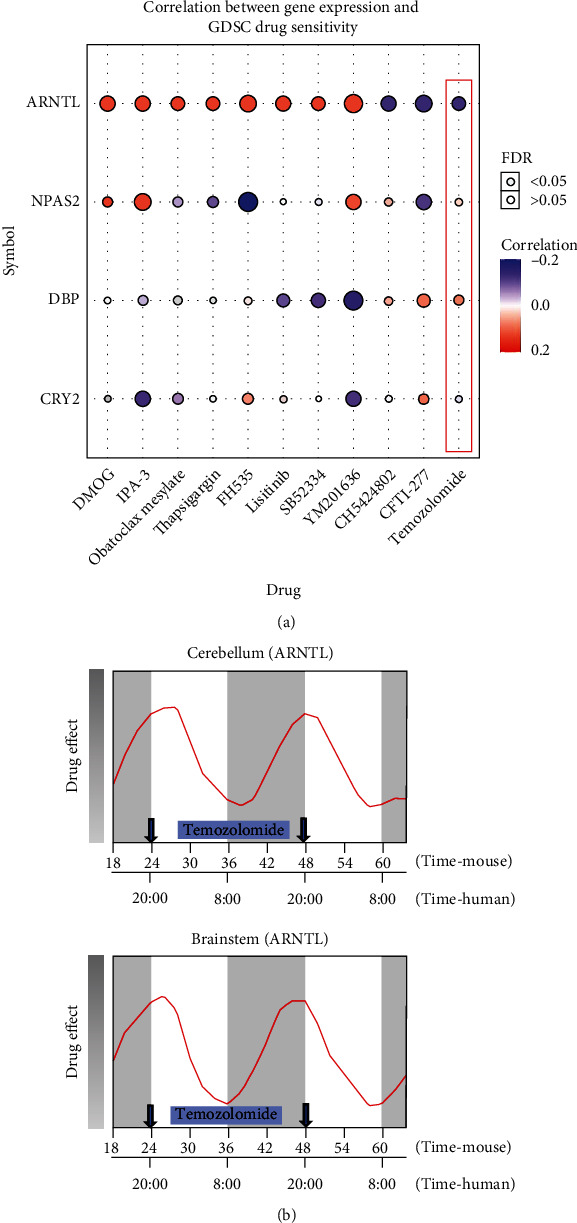
Correlation between the four key CCGs and drug sensitivity. (a) Positive correlation represents that the drug is resistant to high expression of the gene, and vice versa. (b) The drug sensitivity curve of temozolomide varied with the rhythm of *ARNTL* expression.

Drug sensitivity analysis provided a bubble plot of the relationship between the four key CCGs and various cancer drugs. (Figure 11(a)). We used bioinformatics to find the drug sensitivities most associated with the four CCGs in glioma based on GDSC, which is a database containing data on drug sensitivity and gene expression profiles of hundreds of tumor cell lines. The results showed that four CCGs were associated with the sensitivities of several drugs, among which temozolomide was found to be an existing clinical drug for glioma [37]. The blue circle showed negative correlation meant that the higher the expression of *ARNTL*, the lower IC_50_ values (represented higher sensitivity) of temozolomide in cancer cells. Although other CCGs presented similar rhythms, they were not significantly associated with drug sensitivity in the bubble plot. Previous literature suggesting the therapeutic effect of temozolomide on glioma varies with *ARNTL* rhythmic expression, the effect was the best in peak and the worst in the trough [38]. Accordingly, the time-of-temozolomide sensitivity curve was plotted (Figure 11(b)), which clearly demonstrated the different therapeutic potentials of temozolomide at different time points of administration.

## 4. Discussion

In this study, we systematically analyzed and explored the role of CCGs in the diagnosis, prognosis, and chronotherapy of glioma. Key CCGs, including *ARNTL*, *NPAS2*, *CRY2*, and *DBP*, acted as potent diagnosis and prognosis biomarkers of glioma patients and rhythmically regulated cancer-related signaling pathways and coexpressed genes to affect drug sensitivity and possible clinic outcomes. The CCG-based predictive model demonstrated higher predictive accuracy than that of the traditional grade-based model; this new prediction model can greatly improve the accuracy of glioma prognosis. Another study also confirmed the ability of CCGs as prognostic markers for gliomas, suggesting that CCGs' expression affect immunity and cell cycle [22]. However, the unique rhythm expression of CCGs has not been analyzed in the paper. Our study found that the oscillating expression of CCGs has a great impact on diagnosis and prognosis. Rhythmic expression was firstly introduced into the CCG-based predictive model. Appropriate sampling time could greatly improve the ability of early diagnosis and obtain a better prognosis, while inappropriate sampling time may lead to misdiagnosis and delay treatment. In addition, we also predicted the optimal administration time of temozolomide, which provides an effective reference for the chronotherapy of glioma.

The differential expression of CCGs in glioma grading confirmed that cluster I genes (*ARNRL*, *NPAS2*, and *TIMELESS*) and *CRY1,* with cluster II genes (*CRY2*, *DBP*, *NR1D1*, *NR1D2*, *PER2*, *PER3*, and *RORA*) showed significantly opposite expression trends in brain tissues (Figure 3). Besides, the expression of eight CCGs (*CRY2*, *DBP, NR1D1*, *NR1D2*, *PER2*, *PER3*, *RORA*, and *TIMELESS*) was negatively regulated by methylation and positively regulated by CNV in glioma, which is consisting with previous studies that DNA methylation and CNV can promote abnormal gene expression [39–41]. Furthermore, we found that single CCG has high independent predictive accuracy and may be a potential prognostic biomarker for glioma (Figures 5 and 6). Our CCG-based prediction model had higher accuracy than the traditional grade-based model (Figures 7(a) and 7(b)) [42].

Four key CCGs, including *ARNTL*, *NPAS2*, *CRY2*, and *DBP*, showed opposite behavior in differential expression and prognosis (Figures 7(c) and 7(d)). Previous studies have also confirmed the opposite effects of *NPAS2* and *CRY2* on cancer prognosis [43]. Moreover, the four key CCGs-based risk scores varied along the expression fluctuations. Patients sampled at different times had great impacts on the total predictive points and survival rates (Figure 7(e)). A recent study also indicated that *ARNTL* and *PER1* showed peaks of expression in mice in the morning and evening, respectively, whereas in baboon, the peak phases occurred in opposite time point [35]. Therefore, it was reductive that sampling glioma patients at beginning in the evening (about 8:00 pm) could obtain higher predictive sensibility whereas sampling in the morning might result in a misleading diagnosis.

In addition, the four key CCGs were related to several pathways, including cell cycle, DNA damage, Wnt, MAPK, and mTOR pathways (Figure 8(b)), which was consistent with previous reports showing that *NPAS2* activated MAPK signals to be associated with poor survival of primary tumors [44, 45]. *CRY2* has also been demonstrated to activate the mTOR pathway to regulate the differentiation and function of immune cells [46]. *ARNTL* activated Wnt to promote tumor cells development [47]. On the contrary, *CRY2* and *DBP* inhibited DNA damage and cell cycle pathways to enhance tumor cell growth. These results implied that abnormal expression of CCGs could affect cancer pathways to promote glioma progression. Besides, several genes involved in these pathways were also rhythmically expressed similar to CCGs (Figure 8(c)). Among closely coexpressed genes, the rhythmic expression of *TEF* was exactly the same as that of cluster II genes. *TEF* was controlled by the circadian rhythm and affected the expression of other rhythmic and functional genes [48], suggesting the modulation of TFE-mediated downstream effectors by *CRY2* and *DBP*. What is more, due to correlation of *CRY2* and immune cells, immune infiltration was mainly contributed by *CRY2*, thus influencing the occurrence and progression of glioma.

In general, not all drugs are suitable for chronotherapy, most chronotherapy focuses on drugs with a half-life of less than 15 hours [49]. The plasma concentration of temozolomide peaks within 1 hour (*T*max < 1 h) after oral dosing and has a plasma half-life of 1.8 h, which makes the drug suitable for chronotherapy [49–51]. In fact, previous studies have shown that temozolomide is most effective on glioma cells at the *ARNTL* peak and least effective at the trough [38]. When considering the critical roles of pharmacokinetics and pharmacodynamics in drug metabolism and therapy, it is reasonable that administrating temozolomide at about 7-8:00 pm is likely to obtain a better curative effect. In our work, we showed the therapeutic relationship between temozolomide and *ARNTL* and pointed out a better time to administer temozolomide was about 7-8:00 pm (the peak expression of *ARNTL*) in the evening (Figure 11(b)), suggesting precise chronotherapy for glioma patients.

## 5. Conclusion

In summary, we provided a CCGs-based accurate prediction model and showed that *ARNTL*, *NPAS2*, *CRY2*, and *DBP* had great effects on diagnosis and prognosis of glioma. Besides, if the patient was sampled at night (means to have a higher expression of CCG), it can lead to early diagnosis of glioma, whereas sampling in the morning may cause misdiagnosis and thus delay the treatment of glioma. Furthermore, according to expression of *ARNTL*, appropriate timing of temozolomide administration can effectively improve the efficacy, which provides a reference for chronotherapy of glioma. Our works strengthen the importance of CCGs as prognosis biomarkers and the clinic implications for glioma chronotherapy.

## Figures and Tables

**Table 1:** Univariate analysis and multivariate analysis of clinicopathological characteristics and CCGs for overall survival in glioma patients from TCGA.

Variables	Total *n* = 628	Univariate analysis		Multivariate analysis	
	*n* (%)	HR (95% CI)	*P*	HR (95% CI)	*P*
*Age*					
<60	483 (76.9%)	1 (reference)		1 (reference)	
≥60	145 (23.1%)	1.073 (1.062-1.085)	^∗∗∗^	1.047 (1.034-1.062)	^∗∗∗^
*Gender*					
Female	270 (43.0%)	1 (reference)			
Male	358 (57.0%)	1.08 (0.821-1.419)			
*Grade*					
G2+G3	476 (75.8%)	1 (reference)		1 (reference)	
G4	152 (24.2%)	9.553 (7.036-12.97)	^∗∗∗^	1.802 (1.168-2.779)	^∗∗^
*ARNTL*					
Low exp	336 (53.5%)	1 (reference)		1 (reference)	
High exp	292 (46.5%)	2.612 (2.165-3.145)	^∗∗∗^	1.525 (1.219-1.908)	^∗∗∗^
*CLOCK*					
Low exp	304 (48.4%)	1 (reference)			
High exp	324 (51.6%)	0.565 (0.440-0.727)	^∗∗∗^		
*CRY1*					
Low exp	329 (52.4%)	1 (reference)			
High exp	299 (47.6%)	2.102 (1.555-2.842)	^∗∗∗^		
*CRY2*					
Low exp	274(43.6%)	1 (reference)		1 (reference)	
High exp	354(56.4%)	0.426 (0.375-0.484)	^∗∗∗^	0.666 (0.522-0.85)	^∗∗^
*DBP*					
Low exp	293 (46.6%)	1 (reference)			
High exp	335 (53.4%)	0.49 (0.416-0.576)	^∗∗∗^		
*NPAS2*					
Low exp	319 (50.8%)	1 (reference)		1 (reference)	
High exp	309 (49.2%)	1.431 (1.237-1.654)	^∗∗∗^	1.234[1.034-1.472]	^∗^
*NR1D1*					
Low exp	306 (48.7%)	1 (reference)			
High exp	322 (51.3%)	0.613 (0.526-0.713)	^∗∗∗^		
*NR1D2*					
Low exp	271 (43.2%)	1 (reference)			
High exp	357 (56.8%)	0.352 (0.297-0.417)	^∗∗∗^		
*PER1*					
Low exp	275 (43.8%)	1 (reference)			
High exp	353 (56.2%)	0.853 (0.737-0.987)	^∗^		
*PER2*					
Low exp	294 (46.8%)	1 (reference)			
High exp	334 (53.2%)	0.464 (0.385-0.559)	^∗∗∗^		
*PER3*					
Low exp	285 (45.4%)	1 (reference)			
High exp	343 (54.6%)	0.489 (0.434-0.551)	^∗∗∗^		
*RORA*					
Low exp	297 (47.3%)	1 (reference)		1 (reference)	
High exp	331 (52.7%)	0.584 (0.498-0.685)	^∗∗∗^	0.745 (0.566-0.981)	^∗^
*TIMELESS*					
Low exp	343 (54.6%)	1 (reference)		1 (reference)	
High exp	285 (45.4%)	2.051 (1.775-2.369)	^∗∗∗^	1.39 (1.132-1.707)	^∗∗^

**Table 2:** Univariate analysis and multivariate analysis of clinicopathological characteristics and CCGs for overall survival in glioma patients from CGGA.

Variables	Total *n* = 961	Univariate analysis		Multivariate analysis	
	*n* (%)	HR (95% CI)	*P*	HR (95% CI)	*P*
*Age*					
<60	847 (88.1%)	1 (reference)		1 (reference)	
≥60	113 (11.9%)	1.029 (1.022-1.036)	^∗∗∗^	1.015 (1.008-1.022)	^∗∗∗^
*Gender*					
Female	397 (41.3%)	1 (reference)			
Male	564 (58.7%)	1.015 (0.862-1.194)			
*Grade*					
G2+G3	590 (61.4%)	1 (reference)		1 (reference)	
G4	371 (38.6%)	4.151 (3.509-4.912)	^∗∗∗^	1.971 (1.600-2.429)	^∗∗∗^
*ARNTL*					
Low exp	450 (46.8%)	1 (reference)			
High exp	511 (53.2%)	1.282 (1.173-1.4)	^∗∗∗^		
*CLOCK*					
Low exp	510 (53.1%)	1 (reference)			
High exp	451 (46.9%)	1.208 (1.080-1.352)	^∗∗∗^		
*CRY1*					
Low exp	466 (48.5%)	1 (reference)			
High exp	495 (51.5%)	1.338 (1.225-1.463)	^∗∗∗^		
*CRY2*					
Low exp	467(48.6%)	1 (reference)		1 (reference)	
High exp	494 (51.4%)	0.484 (0.441-0.53)	^∗∗∗^	0.679 (0.582-0.793)	^∗∗∗^
*DBP*					
Low exp	447 (46.5%)	1 (reference)		1 (reference)	
High exp	514 (53.5%)	0.763 (0.714-0.816)	^∗∗∗^	0.872 (0.775-0.981)	^∗^
*NPAS2*					
Low exp	489 (50.9%)	1 (reference)			
High exp	472 (49.1%)	1.317 (1.204-1.441)	^∗∗∗^		
*NR1D1*					
Low exp	436 (45.4%)	1 (reference)			
High exp	525 (54.6%)	0.834 (0.773-0.899)	^∗∗∗^		
*NR1D2*					
Low exp	471 (49.0%)	1 (reference)			
High exp	490 (51.0%)	0.716 (0.649-0.789)	^∗∗∗^		
*PER1*					
Low exp	433 (45.1%)	1 (reference)			
High exp	528 (54.9%)	1.007 (0.943-1.074)			
*PER2*					
Low exp	495 (51.5%)	1 (reference)			
High exp	466 (48.5%)	0.66 (0.562-0.774)	^∗∗∗^		
*PER3*					
Low exp	486 (50.6%)	1 (reference)			
High exp	475 (49.4%)	0.767 (0.703-0.836)	^∗∗∗^		
*RORA*					
Low exp	465 (48.4%)	1 (reference)		1 (reference)	
High exp	496 (51.6%)	0.842 (0.745-0.952)	^∗∗^	0.854 (0.731-0.999)	^∗^
*TIMELESS*					
Low exp	513 (53.4%)	1 (reference)		1 (reference)	
High exp	448 (46.6%)	1.722 (1.595-1.859)	^∗∗∗^	1.335 (1.196-1.491)	^∗∗∗^

Notes: characteristics with *P* < 0.05 in the univariate analysis were further screened in the multivariate analysis. Exp: expression; HR: hazard ratio; CI: confidence interval; ^∗^*P* < 0.05, ^∗∗^*P* < 0.01, and ^∗∗∗^*P* < 0.001.

**Table 3:** Correlation analysis between four key CCGs and immune cell biomarkers for glioma.

Immune cells	Biomarkers	ARNTL		NPAS2		CRY2		DBP	
		*P*	Cor	*P*	Cor	*P*	Cor	*P*	Cor
CD8 + T cell	CD8A	^∗∗∗^	0.14	^∗∗∗^	0.31	^∗∗∗^	-0.27	^∗∗^	-0.09
	CD8B	^∗∗∗^	0.16	^∗∗∗^	0.22	^∗∗∗^	-0.32	^∗∗^	-0.11
T cell (general)	CD3D	^∗∗∗^	0.13	^∗∗∗^	0.17	^∗∗∗^	-0.37	^∗∗^	-0.12
	CD3E	^∗^	0.09	^∗∗∗^	0.22	^∗∗∗^	-0.34	^∗∗^	-0.1
	CD2	^∗∗∗^	0.14	^∗∗∗^	0.21	^∗∗∗^	-0.37	^∗∗∗^	-0.13
B cell	CD19	0.99	0	0.31	0.03	^∗∗∗^	-0.16	0.78	0.01
	CD79A	0.84	0.01	0.69	0.01	^∗∗∗^	-0.15	0.66	-0.01
Tumor-associated macrophage (TAM)	CCL2	^∗∗∗^	0.15	^∗∗∗^	0.17	^∗∗∗^	-0.24	^∗∗∗^	-0.13
	CD68	0.07	0.07	^∗^	0.09	^∗∗∗^	-0.4	^∗∗∗^	-0.15
	IL10	^∗∗∗^	0.15	^∗∗∗^	0.12	^∗∗∗^	-0.33	^∗∗∗^	-0.18
Neutrophils	CD66b (CEACAM8)	0.4	0.03	0.98	0	^∗^	-0.08	0.15	-0.05
	CD11b (ITGAM)	0.35	0.03	^∗∗∗^	0.12	^∗∗∗^	-0.36	0.39	-0.03
	CCR7	0.13	0.06	^∗∗∗^	0.16	^∗∗∗^	-0.23	^∗∗∗^	-0.13
Natural killer cell	KIR2DL4	0.65	0.02	0.72	0.01	^∗∗∗^	-0.23	0.32	-0.03
	KIR3DL1	0.06	0.07	^∗∗∗^	0.13	0.05	-0.07	^∗^	-0.09
	KIR2DS4	0.34	0.03	0.08	0.06	^∗∗∗^	-0.17	^∗^	-0.07
Dendritic cell	HLA-DPB1	^∗∗^	0.11	^∗∗∗^	0.13	^∗∗∗^	-0.44	^∗∗^	-0.1
	HLA-DQB1	^∗∗^	0.15	^∗∗∗^	0.12	^∗∗∗^	-0.4	^∗∗∗^	-0.15
	HLA-DRA	^∗∗∗^	0.19	^∗∗∗^	0.13	^∗∗∗^	-0.49	^∗∗∗^	-0.19
	HLA-DPA1	^∗∗∗^	0.13	^∗∗∗^	0.13	^∗∗∗^	-0.44	^∗∗∗^	-0.17
	BDCA-1 (CD1C)	0.98	0	0.39	0.03	^∗∗∗^	-0.14	0.24	-0.04
	BDCA-4 (NRP1)	^∗∗∗^	0.2	^∗∗∗^	0.44	^∗∗∗^	-0.4	^∗∗∗^	-0.31
	CD11c (ITGAX)	0.24	-0.04	0.92	0	^∗∗∗^	-0.33	0.11	0.059

Notes: Cor: *R* value of Person's correlation; ^∗∗∗^*P* < 0.05, ^∗∗^*P* < 0.01, and ^∗^*P* < 0.001.

## Data Availability

The datasets presented in this study can be found in online datasets, including TCGA (https://cancergenome.nih.gov), CGGA (http://www.cgga.org.cn/), and GEO databases (https://www.ncbi.nlm.nih.gov/geo).

## Abbreviations

TCGA: The Cancer Genome Atlas
CGGA: Chinese Glioma Genome Atlas
KIRC: Kidney renal clear cell carcinoma
CCG: Circadian clock gene
DEG: Differentially expressed gene
AUC: Area under curve
FDR: False discovery rate
HR: Hazard ratio
ROC: Receiver operating characteristic.

## Supplementary Materials

Supplementary Materials Result of ROC analysis of each grade of glioma in TCGA and CGGA were in Supplementary Materials (Figure S1).
