# Supplementary material for: Circadian Clock Genes Act as Diagnostic and Prognostic Biomarkers of Glioma: Clinic Implications for Chronotherapy
Source: Biomed Res Int. 2022 Jul 4;2022:9774879. doi: 10.1155/2022/9774879 (PMC9273445; doi:10.1155/2022/9774879)
Supplement: Supplementary Materials — Result of ROC analysis of each grade of glioma in TCGA and CGGA were in Supplementary Materials (Figure S1). [file 9774879.f1.pdf]

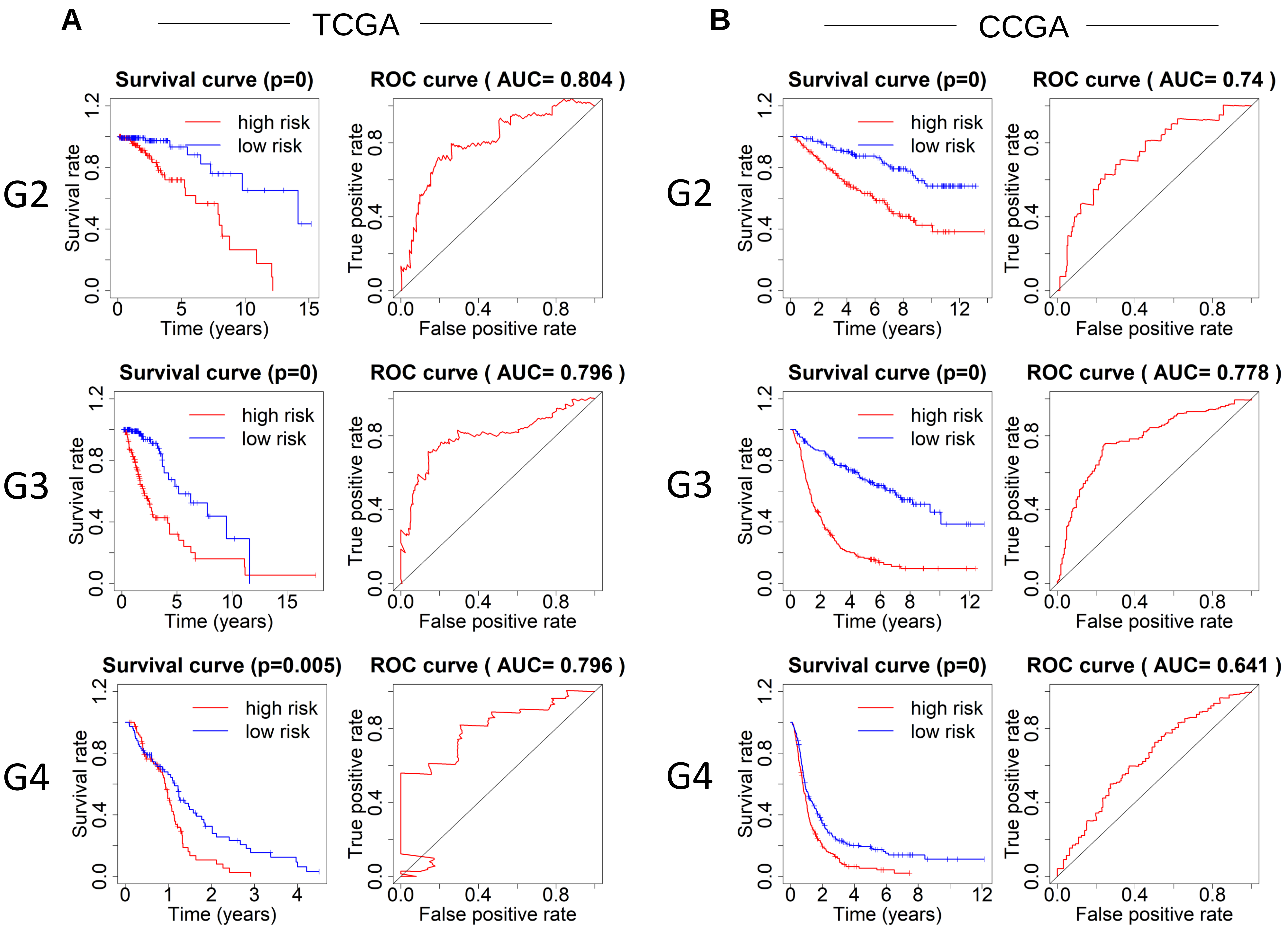

Figure S1 (A) ROC analysis of each grade of glioma in TCGA showed similar AUC values in G2~G4. (B) ROC analysis of each grade of glioma in CGGA showed similar AUC values in G2 and G3.
